# Beyond PD-1/PD-L1: Reprogramming the Gynecologic Tumor Microenvironment by Targeting TIGIT and Myeloid Suppression

**DOI:** 10.3390/ijms27125373

**Published:** 2026-06-14

**Authors:** Shanza Waseem, Jun Zhan, Xue Xiao

**Affiliations:** 1 Department of Gynecology and Obstetrics, West China Second University Hospital, Sichuan University, Chengdu 610041, China; 2 Key Laboratory of Birth Defects and Related Diseases of Women and Children (Sichuan University), Ministry of Education, West China Second Hospital, Sichuan University, Chengdu 610041, China; 3 Tianfu Jincheng Laboratory, Chengdu 610041, China; 4 Laboratory of Stem Cell &Embryo Development, West China Second Hospital, Sichuan University, Chengdu 610041, China

**Keywords:** gynecologic oncology, tumor immunoediting, immune checkpoint inhibitors, TIGIT, myeloid-derived suppressor cells, regulatory T cells, tumor microenvironment, translational immunotherapy

## Abstract

Immune checkpoint inhibitors targeting the PD-1 (Programmed Cell Death Protein 1)/PD-L1 (Programmed Death-Ligand 1) axis have transformed cancer therapeutics, yet their efficacy in gynecologic malignancies particularly high-grade serous ovarian carcinoma remains disappointingly limited. This therapeutic resistance stems from a highly orchestrated, multidimensional immunosuppressive tumor microenvironment (TME) characterized by the convergent actions of regulatory T cells (Tregs), myeloid-derived suppressor cells (MDSCs), and an inhibitory cytokine network (IL-10, TGF-β, VEGF). Emerging evidence positions TIGIT (T-cell immunoreceptor with immunoglobulin and ITIM domain) as a master checkpoint integrator that coordinately regulates CD8+ T-cell exhaustion, NK-cell dysfunction, and Treg-mediated suppression. Dual blockade of PD-1 and TIGIT represents a mechanistically rational strategy to dismantle this immunosuppressive fortress. This review synthesizes current understanding of the gynecologic TME architecture, delineates the molecular and cellular basis for TIGIT/PD-1 synergy, critically evaluates ongoing clinical translation efforts, and proposes an integrative framework leveraging spatial transcriptomics, single-cell resolution immunoprofiling, and patient-derived experimental models to accelerate biomarker-driven therapeutic development.

## Simple Summary

Immunotherapy drugs that block PD-1/PD-L1 have transformed cancer treatment, but they show limited success in gynecologic cancers such as ovarian, endometrial, and cervical malignancies. This resistance occurs because these tumors create a powerful protective barrier of suppressive immune cells and signals that exhaust the body’s cancer-fighting lymphocytes. This review examines a promising new strategy that combines PD-1 blockade with drugs targeting TIGIT, a “master switch” that simultaneously controls exhausted T cells, natural killer cells, and regulatory T cells. We describe how these two checkpoints work together to suppress anti-tumor immunity and summarize the ongoing clinical trials evaluating this combination in gynecologic cancers. We also discuss the major challenges facing this approach, including tumor heterogeneity and biomarker development, and propose an integrated framework using advanced technologies to accelerate personalized immunotherapy for women with gynecologic malignancies.

## 1. Introduction: The Immunotherapeutic Impasse in Gynecologic Malignancies

Immune checkpoint inhibitors targeting PD-1 and PD-L1 have truly revolutionized how we treat many solid tumors, offering durable responses where conventional therapies once fell short. Yet when we turn our attention to gynecologic cancers, the picture becomes considerably more complicated, and in ovarian cancer, the results have been largely disappointing. Across multiple randomized trials in advanced ovarian cancer, adding anti-PD-1 or anti-PD-L1 therapy has failed to meaningfully extend progression-free survival compared to standard treatment, with a pooled hazard ratio hovering around 0.98. Neither first-line nor recurrent settings have shown clear benefit, and even patients selected for PD-L1 positivity or homologous recombination deficiency have not fared better, highlighting an urgent need for smarter biomarker strategies and rationally designed combination approaches [1]. Multiple analyses point to the tumor’s immunosuppressive microenvironment, generally low mutational burden, and inconsistent T-cell infiltration as key culprits; with monotherapy response rates stubbornly low, the field has pivoted toward combining checkpoint inhibitors with chemotherapy, PARP inhibitors, and anti-angiogenic agents though consistent survival gains remain elusive [2].

Looking across the spectrum of gynecologic malignancies, PD-1/PD-L1 monotherapy performs quite differently depending on tumor type and genomic context. In recurrent or metastatic cervical cancer, pembrolizumab as a single agent achieves objective response rates around 12% in PD-L1-positive tumors, which was enough to secure regulatory approval for previously treated patients [3]. Endometrial cancer tells two distinct stories: tumors with mismatch repair deficiency (dMMR) or microsatellite instability-high (MSI-H) representing about a quarter to a third of cases respond favorably to PD-1 blockade, with response rates ranging from 45% to 57% across different agents. But for the remaining patients with proficient mismatch repair (pMMR) or microsatellite stable (MSS) disease, monotherapy activity is modest at best, typically landing between 3% and 10% [4].

High-grade serous ovarian carcinoma (HGSOC) stands out as particularly resistant to PD-1/PD-L1 monotherapy, despite compelling preclinical rationale. Response rates in early-phase trials and real-world analyses consistently fall below 10–15%, reflecting the low prevalence of MSI-H and generally modest PD-L1 expression and tumor mutational burden in ovarian cancer [5]. These patterns have shaped current treatment strategies, which prioritize single-agent PD-1 inhibitors for MSI-H/dMMR endometrial cancer and biomarker-selected cervical cancer, while combination approaches are being aggressively pursued to overcome primary resistance in MSS endometrial cancer and HGSOC [4].

What this differential responsiveness teaches us is that effective immunotherapy demands more than simply reversing T-cell exhaustion it requires fundamentally reshaping the complex ecosystem within which tumors evolve, adapt, and spread [6,7].

The tumor microenvironment in gynecologic cancers operates as a sophisticated, multilayered system of immune privilege. Malignant cells cleverly co-opt multiple cellular lineages including regulatory T cells, tumor-associated macrophages, and cancer-associated fibroblasts alongside soluble mediators like cytokines, chemokines, lipids, and extracellular vesicles to establish durable immunological tolerance that sustains tumor growth, metastasis, and resistance to therapy [8,9].

Central to this immunosuppressive network, as highlighted in this Research Topic, are dense infiltrates of FoxP3+ regulatory T cells (Tregs) and accumulating myeloid-derived suppressor cells (MDSCs). These populations work together to create an inhibitory cytokine environment rich in interleukin-10 (IL-10), transforming growth factor-beta (TGF-β), and vascular endothelial growth factor (VEGF). Together, they suppress dendritic cell maturation, expand Treg populations, and choke off effector T-cell function in gynecologic malignancies [10]. In ovarian and endometrial cancers, elevated IL-10 and TGF-β levels track closely with increased Treg infiltration and worse survival outcomes, while VEGF expression further stabilizes immune tolerance and impairs antigen presentation underscoring how these factors cooperatively sustain a suppressive tumor microenvironment [11].

The field has therefore shifted toward rational combination strategies designed to hit multiple, non-redundant immunosuppressive pathways simultaneously. Among the most promising approaches is dual blockade of PD-1 and TIGIT (T-cell immunoreceptor with immunoglobulin and ITIM domain). These two checkpoints are frequently co-expressed on exhausted T cells and exert complementary control over CD28/CD226 co-stimulation, helping to preserve stem-like, tumor-reactive T-cell clones while limiting terminal exhaustion. Preclinical models and early clinical testing suggest this combination enhances antitumor efficacy [12]. Early human studies indicate that co-inhibition can improve response rates and progression-free survival across several solid tumors compared to historical anti-PD-1 monotherapy data, with mechanistic work revealing that PD-1/TIGIT co-blockade promotes CD226-driven clonal expansion and functional reinvigoration of cytotoxic T cells effects that may be particularly pronounced in PD-L1-positive disease contexts [13].

TIGIT has emerged as a particularly attractive therapeutic target because it sits at a unique intersection of adaptive and innate immune regulation, simultaneously modulating CD8+ T-cell effector function, natural killer (NK) cell cytotoxicity, and regulatory T-cell suppressive capacity [14]. Through engagement with the CD155 axis and competition with the activating receptor CD226, TIGIT dampens cytotoxicity and cytokine production in both CD8+ T cells and NK cells while stabilizing Treg-mediated suppression. This triple effect collectively enforces an immunosuppressive tumor microenvironment that checkpoint blockade can potentially reverse [15].

In this review, we aim to synthesize current understanding of immune evasion in gynecologic cancers, with particular attention to the mechanistic rationale supporting TIGIT/PD-1 co-targeting. We critically examine the translational challenges that continue to slow clinical progress including spatial and temporal heterogeneity, persistent biomarker limitations, and inadequacies in preclinical models and propose an integrated methodological framework to accelerate development of personalized immunotherapeutic strategies.

## 2. The Multidimensional Architecture of Immune Evasion in Gynecologic Cancers

Single-agent checkpoint blockade fails in most gynecologic tumors because these cancers employ parallel, compensatory immunosuppressive mechanisms that collectively erect a formidable barrier to anti-tumor immunity [16].

Within the gynecologic tumor microenvironment, suppressive networks converge. Regulatory T cells (Tregs), myeloid-derived suppressor cells (MDSCs), and tumor-associated macrophages coordinate with metabolic cues such as lactate and checkpoint upregulation to blunt effector T-cell function and sustain exhaustion. This layered suppression explains why PD-1/PD-L1 monotherapy proves insufficient [17].

Lactate exemplifies this complexity: elevated levels preferentially upregulate PD-1 on Tregs, paradoxically enhancing their suppressive activity during PD-1 blockade and contributing to treatment failure. Such compensatory mechanisms underscore why single-axis inhibition rarely succeeds and why rational combination strategies are essential to dismantle these redundant suppressive circuits [17].

### 2.1. Cellular Hubs of Immunosuppression

#### 2.1.1. Regulatory T Cells: Specialized Architects of Immune Tolerance

Regulatory T cells (Tregs), identified by FoxP3 expression, serve as master architects of immune suppression in gynecologic malignancies. In high-grade serous ovarian carcinoma (HGSOC), single-cell and flow cytometry studies reveal marked Treg enrichment within both solid tumors and malignant ascites. These cells constitute a dominant CD4+ subset in the tumor microenvironment compared to peripheral blood, exhibiting functional activation and proliferation states that actively reinforce local immune suppression [18,19]. Elevated FoxP3 expression and higher Treg burden consistently predict worse clinical outcomes. Patients in the highest FoxP3-expression strata experience substantially shorter overall survival (27.8 vs. 77.3 months) and progression-free survival (18.0 vs. 57.5 months), demonstrating that Treg accumulation carries real clinical consequence rather than mere correlative significance [20]. Large cohort analyses further emphasize that the effector-to-suppressor balance matters critically: higher CD8/Treg and CD4/Treg ratios independently associate with improved overall survival (hazard ratios 0.84 and 0.88, respectively), underscoring how Treg predominance blunts anti-tumor immunity and diminishes therapeutic responsiveness [21].

The suppressive machinery deployed by Tregs operates through multiple mechanisms: First, cytokine-mediated suppression: Tregs constitutively secrete IL-10 and TGF-β, which exert pleiotropic inhibitory effects on effector T cells and antigen-presenting cells. IL-10 directly suppresses Th1 polarization and dendritic cell maturation, while TGF-β promotes Treg differentiation creating a positive feedback loop and inhibits CD8+ T-cell cytotoxicity [22]. In ovarian cancer, tumor-infiltrating Tregs show upregulated IL-10 and TGF-β expression, which correlates with reduced IFN-γ in CD8+ T cells and impaired CD8+ activation. Notably, this suppression reverses when IL-10 is blocked, supporting a mechanistic role for these cytokines in immune evasion [20,22]. Second, metabolic disruption: Tregs express high levels of CD25 (the IL-2 receptor alpha chain), enabling high-affinity IL-2 capture and competitive consumption that limits IL-2 availability to effector T cells within the tumor microenvironment. This creates an “IL-2 sink” that blunts effector survival and proliferation [23]. By modulating IL-2 homeostasis, Tregs restrict CD8+ effector differentiation and expansion in vivo; conversely, effector-derived IL-2 paradoxically fuels Treg proliferation, reinforcing suppression under IL-2-limited conditions [24]. Third, checkpoint receptor expression: Tregs constitutively express high levels of CTLA-4 and TIGIT compared with conventional T cells, aligning with their enhanced suppressive phenotype and function within tissues and tumors [25]. Additionally, TIGIT marks a particularly suppressive subset of FoxP3+ regulatory T cells with enhanced functional capacity and inhibitory receptor programs, distinguishing them from conventional T cells [26]. CTLA-4 on Tregs directly removes the co-stimulatory ligands CD80/CD86 from antigen-presenting cells via trans-endocytosis, reducing CD28 co-stimulation available to effector T cells and dampening their activation [27]. This physical extraction and depletion of CD80/CD86 at the immune synapse functionally deprives effector T cells of Signal 2 while shifting APCs toward an inhibitory PD-L1-high state that further restrains PD-1-positive effectors [27]. TIGIT marks a highly suppressive subset of FoxP3+ regulatory T cells. TIGIT+ Tregs demonstrate greater suppressive potency than their TIGIT− counterparts and are enriched for effector molecules including IL-10 and CTLA-4, with evidence that their suppression is IL-10-dependent [25]. Transcriptional and functional profiling links TIGIT expression to an activated, lineage-stable Treg phenotype with enhanced suppressive capacity [28]. TIGIT engagement on Tregs activates a distinct suppressive module that increases IL-10 and Fgl2 while selectively dampening pro-inflammatory Th1 and Th17 programs, indicating a TIGIT-driven pathway that extends beyond canonical FoxP3 dependence [25].

#### 2.1.2. Myeloid-Derived Suppressor Cells: The Innate Immune Barrier

Myeloid-derived suppressor cells represent a heterogeneous population of immature myeloid cells that expand pathologically under conditions of chronic inflammation and cancer, where they impair T-cell and natural killer cell function to promote immune evasion [29]. In gynecologic malignancies, both circulating and tumor-infiltrating MDSCs are consistently elevated, with enrichment of monocytic MDSCs (M-MDSC; CD14+HLA-DRlow/−) and polymorphonuclear MDSCs (PMN-MDSC; CD15+CD66b+Lin−HLA-DR−) detectable within the tumor microenvironment and peripheral blood [30].

Their abundance carries clinical weight, correlating with more advanced stage, resistance to therapy, and poorer outcomes. In ovarian cancer, higher levels of CD14+HLA-DRlow/− M-MDSCs in blood and ascites associate with shorter relapse-free survival, driven by IL-6/IL-10–STAT3 signaling that upregulates ARG1 and iNOS to suppress antitumor immunity [31]. In cervical cancer, elevated Lin−/lowHLA-DR−CD11b+CD33+ MDSCs in blood and tumor correlate with stage and reduced relapse-free survival, and functionally inhibit CD4+ and CD8+ T-cell proliferation and cytokine production underscoring their clinical relevance [32].

MDSCs suppress anti-tumor immunity through multiple convergent mechanisms: First, MDSCs express high levels of arginase-1 (ARG1), which hydrolyzes L-arginine to ornithine and urea. L-arginine depletion within the tumor microenvironment has profound consequences for T-cell function: it downregulates CD3ζ chain expression a critical component of the TCR signaling complex arrests T cells in G0-G1 phase, and impairs memory T-cell formation [33,34]. Second, MDSCs generate copious amounts of reactive oxygen species (ROS) and reactive nitrogen species (RNS), including peroxynitrite. These molecules induce T-cell apoptosis, promote nitration of the TCR complex (altering antigen recognition), and modify chemokines in ways that disrupt T-cell trafficking [35,36]. Third, MDSCs express the xc− cystine/glutamate antiporter, enabling them to import extracellular cystine. However, because they lack the ASC neutral amino acid transporter, they cannot export cysteine creating a critical deprivation for T cells, which cannot import cystine directly and depend on exogenous cysteine from antigen-presenting cells [37]. This cystine uptake by MDSCs reduces the cysteine pool available to T cells, blunting their activation and proliferation and reinforcing immune suppression within the tumor microenvironment [38]. Fourth, MDSCs secrete IL-10 and TGF-β, sustaining an immunosuppressive milieu that suppresses effector T cells while promoting differentiation of regulatory T cells from naive CD4+ precursors via IL-10/TGF-β–dependent pathways [39]. In both infections and cancer, elevated IL-10 and TGF-β from MDSCs correlate with increased FoxP3+ Tregs and type 1 regulatory T (Tr1) cells, reinforcing suppression through reduced proinflammatory cytokines and dampened T-cell activation [40].

### 2.2. The Inhibitory Cytokine Network

The cellular constituents of the immunosuppressive tumor microenvironment operate within a rich soluble milieu that perpetuates and amplifies their suppressive functions. This cytokine network creates a self-reinforcing ecosystem where molecular and cellular players collaborate to maintain immune privilege.

Transforming Growth Factor-β (TGF-β) is abundantly present in the gynecologic tumor microenvironment, produced by cancer cells, cancer-associated fibroblasts, Tregs, and MDSCs. TGF-β exerts context-dependent effects but generally promotes immune exclusion by inhibiting T-cell infiltration, suppressing cytotoxic gene programs including perforin, granzyme, and IFN-γ and driving differentiation of pro-tumorigenic Th17 cells. Beyond immune modulation, TGF-β induces epithelial–mesenchymal transition (EMT), enhancing cancer cell invasiveness and metastatic potential [41,42].

Interleukin-10 (IL-10) predominantly suppresses Th1-type immunity in tumors by inhibiting dendritic cell maturation, leading to reduced IL-12 production and impaired cytotoxic T-cell and natural killer cell activation [43]. It also downregulates MHC class II through suppression of the CIITA type I promoter during dendritic cell differentiation, limiting antigen presentation. Additionally, IL-10 directly inhibits CD4+ T-cell proliferation while promoting anergic, CTLA-4-expressing CD4+ T cells [44].

Vascular Endothelial Growth Factor (VEGF): exerts potent immunosuppressive effects. It blocks dendritic cell differentiation from hematopoietic progenitors, impairing maturation and antigen presentation through VEGFR signaling and NF-κB inhibition, which ultimately reduces T-cell priming [45]. VEGF also drives expansion and recruitment of myeloid-derived suppressor cells, establishing a VEGF–MDSC axis of immune evasion that correlates with fewer intratumoral CD8+ T cells and worse survival in both human and murine models [46].

Furthermore, VEGF induces inhibitory checkpoint programs on tumor-infiltrating T cells; VEGF/VEGFR2 signaling directly modulates T-cell phenotype and function, increasing exhaustion markers such as PD-1 within the tumor microenvironment. This provides a mechanistic rationale for combining anti-VEGF agents with PD-1/PD-L1 blockade [47]. Collectively, these pathways position VEGF as a central mediator of tumor-associated immunosuppression that complements its angiogenic role [48].

### 2.3. Tumor Type-Specific Microenvironmental Landscapes

A critical insight emerging from recent immunogenomic analyses is that the tumor microenvironment composition varies substantially across gynecologic tumor types and even within histologically similar tumors. This heterogeneity has profound implications for therapeutic strategy selection [49,50].

As summarized in Table 1, the immunological landscape varies dramatically across gynecologic malignancies, with inflamed phenotypes in MSI-H endometrial and cervical cancers predicting superior ICI responses compared to the immune-excluded/desert profiles of HGSOC and MSS endometrial tumors [25,28,49,51,52,53,54,55,56,57,58,59,60,61,62,63,64,65,66,67,68,69,70,71,72,73,74,75,76,77,78,79,80,81,82,83,84]. This heterogeneity explains the differential responses to PD-1/PD-L1 blockade and highlights the need for mechanistically informed combination strategies tailored to the dominant immunosuppressive pathways operating in individual tumors [85,86].

## 3. TIGIT: A Master Integrator of Lymphocyte Regulation

The inhibitory receptor TIGIT has emerged as a compelling therapeutic target because it sits at a unique intersection of immune regulation. Unlike PD-1, which primarily constrains T cells exhausted by chronic antigen exposure, TIGIT simultaneously dampens CD8+ T-cell effector function, suppresses natural killer cell cytotoxicity, and enhances regulatory T-cell-mediated inhibition making it a true master integrator of immunosuppressive signals [66].

At the molecular level, TIGIT engages CD155 and CD112 ligands that are commonly overexpressed on tumor cells and antigen-presenting cells within the gynecologic tumor microenvironment. This engagement directly counters the activating receptor DNAM-1 (CD226), creating a competitive dynamic where TIGIT binding disrupts CD226-driven co-stimulation. The result is reduced proliferation, diminished cytokine production, and impaired cytotoxicity in both CD8+ T cells and NK cells [87].

What makes TIGIT particularly attractive as a therapeutic target is this dual role: it simultaneously throttles effector cells while actively potentiating Treg suppressive programs. By operating across both effector and regulatory compartments, TIGIT reinforces immune tolerance from multiple angles, which also means that blocking it could release anti-tumor immunity through several coordinated mechanisms [87].

### 3.1. Molecular Architecture and Ligand Interactions

TIGIT also known as WUCAM, Vstm3, or VSIG9 is a type I transmembrane protein belonging to the poliovirus receptor (PVR)/nectin family. Its extracellular region contains a single immunoglobulin variable-set (IgV) domain, while its cytoplasmic tail harbors two distinct inhibitory signaling motifs: an immunoreceptor tyrosine-based inhibition motif (ITIM) and an immunoglobulin tail tyrosine (ITT)-like motif.

Upon ligand engagement, these motifs recruit adaptor molecules such as Grb2 and SHIP1 to mediate downstream suppression [88]. Functional studies confirm that phosphorylation of the ITT-like and ITIMs following CD155 binding drives negative signaling that blunts cytotoxicity and cytokine production in lymphocytes, consistent with TIGIT’s role as a bona fide immune checkpoint [89].

TIGIT binds with highest affinity to CD155 (also called PVR or Necl-5) and with lower affinity to CD112 (PVRL2 or nectin-2). Both ligands are widely expressed on tumor cells and antigen-presenting cells including dendritic cells and macrophages within the tumor microenvironment, enabling broad inhibitory signaling across T cells and natural killer cells [90].

Critically, CD155 and CD112 are shared ligands for the co-stimulatory receptor CD226 (DNAM-1), which delivers activating signals upon engagement on T cells and NK cells. This creates direct competition between TIGIT and CD226 for ligand occupancy. Mechanistically, TIGIT functionally antagonizes CD226 by outcompeting it for CD155 binding due to substantially higher affinity, thereby suppressing CD226-mediated activation and shifting the balance toward immune inhibition [91].

### 3.2. Mechanistic Pathways of TIGIT-Mediated Suppression

TIGIT suppresses anti-tumor immunity through three distinct, non-redundant mechanisms that collectively reinforce immune tolerance from multiple angles.

#### 3.2.1. Cell-Intrinsic Inhibition of CD8+ T Cells

Upon CD155 engagement, TIGIT’s ITIM and ITT-like tyrosine residues are phosphorylated by Src-family kinases, enabling recruitment of the lipid phosphatase SHIP-1 via Grb2 and β-arrestin2. This recruitment attenuates proximal TCR signaling by prematurely terminating PI3K and MAPK pathways, disrupting downstream NF-κB and AP-1 activation. Functionally, this translates to reduced calcium flux, diminished proliferation, decreased effector cytokine production (IFN-γ, TNF-α, IL-2), and impaired cytotoxic granule polarization and release culminating in markedly blunted cytotoxicity [89].

Mechanistically, β-arrestin2 serves as a critical adaptor, linking phosphorylated TIGIT to SHIP-1, which then suppresses TRAF6 autoubiquitination and downstream NF-κB signaling to curtail IFN-γ production. This pathway reinforces broad inhibition of effector functions following TIGIT ligation [92].

Single-cell and bulk transcriptomic studies reveal that TIGIT marks a distinct, partially overlapping dysfunction state relative to PD-1-defined exhaustion. TIGIT+ CD8+ T cells consistently co-express additional inhibitory receptors such as LAG-3 and TIM-3 while showing reduced effector programs, yet they retain features not fully captured by PD-1 expression alone [67]. Across multiple tumor types and diseased tissues, TIGIT+ CD8+ T cells commonly display downregulation of the co-stimulatory receptor CD226 alongside diminished cytokine production, creating a TIGIT–CD226 imbalance that is repeatedly observed in human tumor-infiltrating lymphocytes and dysfunctional CD8+ populations [66].

#### 3.2.2. Regulation of NK Cell Function

Natural killer cells serve as critical frontline defenders in anti-tumor immunity, particularly for controlling metastasis and eliminating “missing-self” targets that have lost MHC class I expression. In this context, NK cytotoxicity is typically unleashed through activating receptors like DNAM-1 (CD226) engaging CD155/CD112 on tumor cells [93].

TIGIT is expressed on human NK cells and functions as a key inhibitory checkpoint. When TIGIT engages its ligands CD155 or CD112 it directly suppresses NK cytotoxicity through its ITIM, effectively counteracting DNAM-1-mediated activation and limiting the killing of MHC class I-deficient tumor cells [94].

This inhibition operates through two coordinated mechanisms: (1) direct ITIM-dependent recruitment of SHIP-1 suppresses Vav1 phosphorylation and downstream actin remodeling, blunting cytotoxic synapse formation; and (2) high-affinity competition with CD226 for shared ligands diminishes DNAM-1 signaling and effector function [95].

Within the tumor microenvironment, the functional state of NK cells is governed by the expression balance between inhibitory TIGIT and activating CD226. Tumors frequently upregulate CD155, shifting this equilibrium toward inhibition and fostering immune evasion and metastatic fitness [96].

#### 3.2.3. Enhancement of Treg Suppressive Function

TIGIT marks a highly suppressive subset of FoxP3+ regulatory T cells. TIGIT+ Tregs demonstrate greater suppressive potency than their TIGIT− counterparts and are enriched for effector molecules including IL-10 and CTLA-4, with evidence that their suppression is IL-10-dependent [25]. Transcriptional and functional profiling links TIGIT expression to an activated, lineage-stable Treg phenotype with enhanced suppressive capacity [28].

TIGIT engagement on Tregs activates a distinct suppressive module that increases IL-10 and Fgl2 while selectively dampening pro-inflammatory Th1 and Th17 programs indicating a TIGIT-driven pathway that extends beyond canonical FoxP3 dependence [25]. In models where TIGIT is ligated, induced Fgl2 is required for Treg-mediated suppression of effector proliferation, reinforcing that TIGIT signaling programs Treg effector outputs toward IL-10 and Fgl2 dominance [25].

Within inflammatory tissues, TIGIT agonism further augments Treg stability and survival programs, increasing expression of lineage and survival markers consistent with enhanced persistence under the metabolic stress typical of the tumor microenvironment [97]. Collectively, these data support a TIGIT-driven, FoxP3-independent signaling module that boosts IL-10 and Fgl2 while reinforcing Treg durability in hostile environments [25,97].

### 3.3. Reciprocal Regulation and the TIGIT/CD226 Axis

The functional outcome of TIGIT engagement is determined not by TIGIT expression alone but by its balance with the competing co-stimulatory receptor CD226, which shares the same ligands and exerts opposing signals on T cells and NK cells [98,99]. This reciprocal regulation creates a dynamic rheostat that sets the lymphocyte activation threshold: higher TIGIT/CD226 ratios favor inhibition, while dominant CD226 co-stimulation lowers the threshold for activation [98,100].

Under homeostatic conditions, CD226 signals promote lymphocyte activation and effector function. However, within the tumor microenvironment, chronic CD155 engagement drives CD226 downregulation on T cells and NK cells via metalloprotease-dependent shedding and transcriptional repression, while TIGIT is maintained or upregulated. This yields a high TIGIT:CD226 ratio that renders lymphocytes profoundly hyporesponsive [101,102].

**Figure 1:**
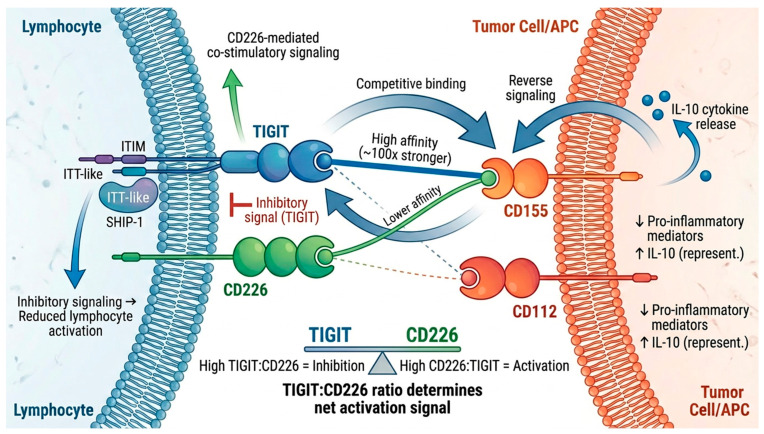
Molecular Architecture and Competitive Dynamics of the TIGIT/CD226/CD155 Checkpoint Axis. The schematic depicts the antagonistic relationship between the inhibitory receptor TIGIT and the co-stimulatory receptor CD226 on lymphocytes (T cells and NK cells). Both receptors compete for the same ligands, CD155 (PVR) and CD112 (Nectin-2), expressed on the surface of tumor cells or antigen-presenting cells (APCs). Green arrows denote CD226-mediated co-stimulatory signaling that promotes effector function, while red blunt-ended arrows indicate TIGIT-mediated inhibitory signaling. Due to its approximately 100-fold higher binding affinity for CD155, TIGIT effectively outcompetes CD226, thereby preventing the activating signals necessary for robust effector function. Upon ligand engagement, TIGIT’s cytoplasmic immunoreceptor tyrosine-based inhibitory motif (ITIM) and immunoglobulin tail tyrosine (ITT)-like motif undergo phosphorylation, recruiting the phosphatase SHIP-1 to attenuate T-cell receptor (TCR) or CD226-mediated signaling. The inset illustrates bidirectional “reverse signaling” in the ligand-bearing cell, promoting a tolerogenic phenotype through the secretion of inhibitory cytokines (e.g., IL-10) and the downregulation of pro-inflammatory mediators. The integration of these signals is determined by the stoichiometric balance of TIGIT and CD226 expression, depicted as a molecular rheostat (balance scale), that regulates immune homeostasis and anti-tumor immunity.

As illustrated in Figure 1, TIGIT’s approximately 100-fold higher binding affinity for CD155 allows it to effectively outcompete CD226 for ligand occupancy. Upon engagement, TIGIT’s cytoplasmic ITIM and ITT-like motifs recruit SHIP-1 to attenuate TCR or CD226-mediated signaling. Concurrently, CD155 engagement can trigger bidirectional “reverse signaling” in ligand-bearing cells, promoting a tolerogenic phenotype through inhibitory cytokine secretion.

Therapeutic TIGIT blockade therefore functions not only by removing inhibitory signals but also by restoring the functional competence of residual CD226 molecules. This dual mechanism inhibition removal and co-stimulation restoration likely underlies the potent synergy observed with PD-1 co-blockade [100,103].

## 4. Mechanistic Rationale for PD-1 and TIGIT Co-Blockade

The complementary and non-redundant functions of PD-1 and TIGIT provide a compelling rationale for their combined targeting. Preclinical studies have demonstrated synergistic anti-tumor efficacy across multiple tumor models, including those resistant to single-agent checkpoint blockade [103,104].

### 4.1. Non-Redundant but Complementary Pathways

PD-1 and TIGIT regulate distinct but intersecting aspects of lymphocyte biology:

**PD-1** primarily functions as a metabolic checkpoint. Upon engagement by PD-L1 or PD-L2, PD-1 recruits the phosphatase SHP-2, which dephosphorylates key TCR-proximal signaling molecules. This results in inhibition of PI3K–Akt signaling, reduced glucose uptake, impaired glycolysis, and promotion of fatty acid oxidation. PD-1 signaling thus enforces a state of metabolic quiescence that limits T-cell proliferation and effector function [105,106].

**TIGIT**, in contrast, primarily regulates cytolytic function and intercellular interactions. Its signaling inhibits the release of cytotoxic granules from CD8+ T cells and NK cells, reduces inflammatory cytokine production, and promotes Treg-mediated suppression. TIGIT also directly competes with CD226 for ligand binding, thereby limiting co-stimulatory input [66,107].

These distinct mechanisms mean that PD-1 blockade alone may restore T-cell proliferation and metabolic fitness but fail to fully restore cytolytic function, particularly in the presence of persistent TIGIT signaling [103]. Conversely, TIGIT blockade may enhance cytotoxic capacity but fail to overcome metabolic exhaustion driven by chronic PD-1 engagement [108].

### 4.2. Overcoming Adaptive Resistance

A critical insight from preclinical models is that PD-1 blockade can induce compensatory upregulation of alternative inhibitory receptors, including TIGIT, as a mechanism of adaptive resistance [66]. Tumors that initially respond to anti-PD-1 therapy but subsequently progress often show increased TIGIT expression on tumor-infiltrating lymphocytes, supporting TIGIT as an acquired resistance pathway that limits the durability of PD-1 monotherapy [109].

Co-blockade of PD-1 and TIGIT preempts this escape route, simultaneously targeting the primary exhaustion pathway (PD-1) and a major compensatory pathway (TIGIT). The resulting therapeutic effect is not merely additive but synergistic, as dual blockade restores CD226 co-stimulation suppressed by both checkpoints and prevents emergence of TIGIT-driven resistance [103,104].

### 4.3. Differential Effects on T-Cell Subsets

Single-cell analyses have revealed that PD-1 and TIGIT are expressed on overlapping but distinct T-cell populations within the tumor microenvironment. PD-1 marks a broader population of exhausted T cells, including terminally exhausted phenotypes characterized by TCF1 loss and TOX upregulation, whereas TIGIT is enriched on more profoundly dysfunctional T cells that co-express multiple inhibitory receptors such as LAG-3 and TIM-3 [110].

Across cancers, most PD-1+ lymphocytes co-express TIGIT (≥90%), but TIGIT expression is relatively more restricted than PD-1 and highlights subsets with deeper exhaustion and higher co-inhibitory receptor load [111].

TIGIT expression on tumor-infiltrating regulatory T cells delineates a highly suppressive subset with elevated FOXP3, CTLA-4, and CD39, enhanced IL-10-dependent suppression, and enrichment within tumors [97,112]. These TIGIT+ Tregs are relatively refractory to signals that destabilize suppression, aligning with observations that PD-1 pathways alone can be insufficient to curb this subset [112].

In melanoma, a high TIGIT/CD226 ratio in Tregs correlates with stronger suppression and poorer outcomes during checkpoint therapy, indicating potential resistance to PD-1-directed strategies [112]. In contrast, TIGIT antagonism reduces intratumoral Tregs and directly weakens their suppressive function in preclinical tumor models, providing a mechanism to counter Treg-mediated resistance that PD-1 blockade does not address [113,114].

### 4.4. Unleashing NK Cell Immunity

NK cells generally express low levels of PD-1, limiting the efficacy of PD-1 blockade in unleashing innate anti-tumor immunity. In contrast, TIGIT is highly expressed on NK cells and functions as a dominant inhibitory checkpoint. TIGIT blockade has been shown to enhance NK cell degranulation, IFN-γ production, and tumor cell killing in multiple preclinical models [115,116].

The contribution of NK cells to anti-tumor immunity is particularly relevant in the context of metastatic disease and in tumors that have downregulated MHC class I to evade T-cell recognition. TIGIT/PD-1 co-blockade thus engages both the adaptive (T-cell) and innate (NK-cell) arms of the immune system, providing broader and more durable tumor control [101,103].

**Figure 2:**
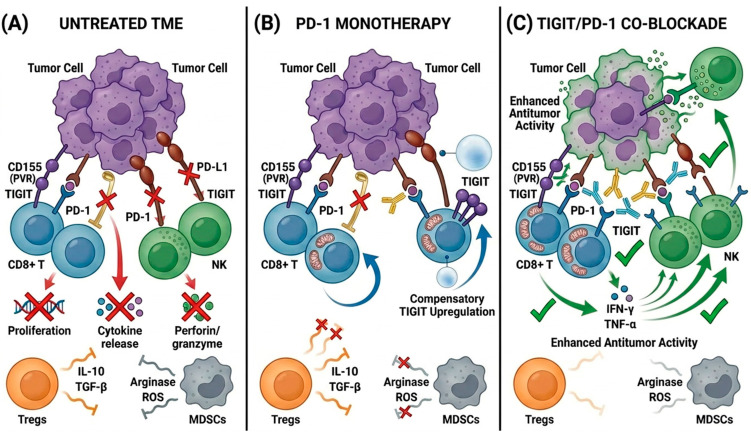
Synergistic Mechanisms of PD-1 and TIGIT Co-Blockade in the Gynecologic Tumor Microenvironment. (**A**) Untreated TME: Tumor cells expressing high levels of CD155 (PVR) and PD-L1 simultaneously engage TIGIT and PD-1 on effector CD8+ T cells and NK cells. TIGIT is also expressed on Tregs, enhancing their suppressive function. This dual engagement delivers parallel inhibitory signals that redundantly suppress lymphocyte proliferation, pro-inflammatory cytokine production (IFN-γ, TNF-α), and cytotoxic granule release (Granzyme B, Perforin). Within this suppressive landscape, TIGIT+ regulatory T cells exhibit enhanced suppressive capacity, secreting IL-10 and TGF-β, while myeloid-derived suppressor cells further inhibit effector functions through arginase and reactive oxygen species. (**B**) PD-1 Monotherapy: Blockade of the PD-1/PD-L1 axis restores T-cell metabolic fitness and proliferative capacity. However, the anti-tumor response remains suboptimal due to persistent TIGIT-mediated inhibitory signaling on CD8+ T cells, NK cells, and TIGIT+ Tregs, which continues to impair cytotoxic effector functions. TIGIT+ Tregs remain highly suppressive, and compensatory upregulation of TIGIT may occur on effector cells as adaptive resistance, limiting response durability. (**C**) TIGIT/PD-1 Co-Blockade: Simultaneous blockade of both pathways synergistically restores CD8+ T-cell metabolic function while maximizing cytotoxic potential. This combination unleashes NK cell activity and significantly attenuates TIGIT+ Treg suppression. By neutralizing two non-redundant inhibitory axes, co-blockade promotes a more robust, polyfunctional, and durable anti-tumor immune response.

As illustrated in Figure 2, the complementary mechanisms of PD-1 and TIGIT blockade work together to remodel the immunosuppressive tumor microenvironment. In the untreated setting (panel A), tumor cells engage both checkpoints simultaneously, suppressing lymphocyte metabolism and cytotoxicity while TIGIT+ Tregs and MDSCs reinforce tolerance. PD-1 monotherapy (panel B) restores metabolic fitness but leaves cytotoxic function constrained by persistent TIGIT signaling and Treg activity. Only dual co-blockade (panel C) fully restores both metabolic and cytotoxic programs while unleashing NK cells and neutralizing Treg-mediated suppression, yielding a polyfunctional anti-tumor response [103,104,105,106,107,108,109,110,111,112,113,114,115,116].

## 5. Translational Landscape: From Preclinical Promise to Clinical Reality

The compelling preclinical rationale for TIGIT/PD-1 co-blockade has prompted rapid clinical translation, with multiple early-phase trials currently evaluating this combination in solid tumors, including gynecologic malignancies [117,118].

As summarized in Table 2, the current clinical trial landscape includes several ongoing studies investigating various anti-TIGIT agents in combination with PD-(L)1 inhibitors across recurrent ovarian, cervical, and endometrial cancers. These trials span phase I/II designs focused on safety, tolerability, and preliminary efficacy signals, with objective response rate and progression-free survival as primary endpoints [119,120,121,122,123,124].

The most clinically advanced agent in this space is tiragolumab, anti-TIGIT monoclonal antibody, which is being evaluated in combination with atezolizumab (anti-PD-L1) across multiple gynecologic cohorts. The phase II NCT03563716 trial is assessing this doublet in recurrent ovarian, cervical, and endometrial cancers, while NCT04354246 adds chemotherapy to the backbone for platinum-sensitive recurrent ovarian cancer [119,120].

Additional programs include AB154 combined with AB122 (anti-PD-1) in advanced solid tumors with gynecologic expansion cohorts [121], vibostolimab partnered with pembrolizumab across various solid tumors [122], and BMS-986207 combined with nivolumab and ipilimumab in recurrent gynecologic cancers [123]. EOS884448 is also being evaluated in early-phase testing with pembrolizumab [124].

The diversity of these approaches from doublet immunotherapy to triplet combinations incorporating chemotherapy reflects the field’s recognition that effective immunotherapy in gynecologic cancers will likely require multifaceted strategies tailored to tumor type and immune contexture.

### 5.1. Emerging Efficacy Signals and Safety Considerations

While randomized efficacy data specifically in gynecologic cancers remain limited, early-phase signals across tumor types support the biological rationale for TIGIT/PD-1 co-blockade. Critically, no randomized Phase III data yet exist for any TIGIT/PD-1 combination in gynecologic malignancies, and the available evidence remains confined to early-phase trials with small sample sizes and exploratory endpoints. The phase II CITYSCAPE study in non-small cell lung cancer provided pivotal proof-of-concept: tiragolumab plus atezolizumab improved objective response rate (31.3% vs. 16.2%) and progression-free survival (hazard ratio 0.57–0.58) compared to atezolizumab alone, demonstrating clear synergy from dual checkpoint inhibition [119]. Subsequent translational work suggests this combination may engage Fcγ receptors to activate macrophages and reprogram exhausted CD8+ T cells, offering mechanistic insight into the observed clinical benefit [125].

In gynecologic populations, early signals are beginning to emerge. A phase I program evaluating the anti-TIGIT antibody AB154 combined with anti-PD-1 AB122 has reported objective activity, including a response in a platinum-resistant ovarian cancer patient previously exposed to checkpoint inhibitors suggesting potential efficacy even in heavily pretreated disease [126]. Expansion cohorts in cervical and endometrial cancers are actively enrolling within broader immunotherapy combination strategies, reflecting the rapidly evolving checkpoint landscape in gynecologic oncology [127].

Encouragingly, the safety profile of TIGIT/PD-1 co-blockade appears manageable and generally similar to PD-1 monotherapy. The most common treatment-emergent adverse events include fatigue, pruritus, rash, and diarrhea consistent with immune-mediated mechanisms. Immune-related events such as pneumonitis, colitis, and hepatitis occur at rates comparable to anti-PD-1 alone, based on early-phase studies of vibostolimab plus pembrolizumab and etigilimab plus nivolumab [117,128].

Critically, no additive toxicity signal has emerged from these phase 1 programs. Grade 3–4 treatment-related adverse events remain low (9–17% with combination therapy) and are dominated by cutaneous and gastrointestinal events typical of PD-1 blockade, without clear evidence of synergistic toxicity from adding TIGIT inhibition. This favorable safety profile supports continued clinical development and ongoing randomized evaluation in gynecologic malignancies [117,128].

### 5.2. Critical Translational Challenges

Despite the compelling biological rationale, significant barriers impede successful translation of TIGIT/PD-1 co-blockade to gynecologic cancers. These challenges span inadequate efficacy data, profound tumor heterogeneity, biomarker complexities, and limitations of preclinical models each demanding careful consideration in trial design and interpretation [13,121].

#### 5.2.1. Spatial and Temporal Heterogeneity

The immune landscape of gynecologic tumors is dynamic and spatially heterogeneous, with adjacent regions ranging from T cell-inflamed “hot” areas to immune-desert zones. This spatial variation arises from differences in ligand expression, stromal barriers, and tumor-intrinsic programs that shape infiltration and exclusion [129,130].

The clinical implications are substantial. Single-site sampling can miss neighboring immune-desert or excluded regions, underestimating immune evasion pathways and leading to misclassification of “hot” tumors that are not uniformly inflamed [131]. In ovarian cancer specifically, single-cell maps reveal distinct immune-infiltrated, excluded, and desert phenotypes within and across lesions, with divergent chemokine networks and antigen-presentation levels reinforcing that one pretreatment biopsy may not capture the overall contexture [130]. Spatial profiling or multi-region assessment, when feasible, significantly improves representation of the tumor immune landscape [130,131].

Temporal heterogeneity proves equally challenging. The tumor microenvironment evolves dynamically under therapeutic pressure, with shifts in immune cell composition, checkpoint molecule expression, and metabolic programming occurring over weeks to months [132,133]. Serial biopsies during treatment are essential for pharmacodynamic assessment but remain difficult to obtain routinely due to invasiveness, cost, and sampling constraints [134,135].

#### 5.2.2. Biomarker Development Challenges

Robust predictive biomarkers for TIGIT/PD-1 co-blockade remain elusive. Unlike PD-L1 which is measured on tumor cells TIGIT is expressed on immune cells, fundamentally complicating quantification and standardization [136]. Several biomarker strategies are under active investigation:

TIGIT expression density: The level of TIGIT on tumor-infiltrating lymphocytes may associate with functional exhaustion and rescue by TIGIT blockade, suggesting potential predictive value. However, clinically validated thresholds are lacking, and optimal subset weighting whether CD8+ T cells, Tregs, or NK cells should be prioritized remains undefined [66,137].

TIGIT:CD226 ratio: Because TIGIT inhibits while CD226 co-stimulates via shared ligands, their balance may integrate signals more faithfully than TIGIT alone. A high TIGIT: CD226 ratio has been linked to greater Treg suppression and poorer checkpoint outcomes, suggesting this ratio may enrich for benefit from TIGIT blockade if CD226 signaling can be preserved [112]. Preclinical data show anti-TIGIT efficacy depends on the presence of CD226hi CD8+ T cells, with TIGIT blockade enhancing CD226 phosphorylation; thus, patients with high TIGIT but adequate CD226 a favorable TIGIT:CD226 ratio may be most responsive [138].

CD155 expression: Tumor CD155 is the key ligand for TIGIT; without it, TIGIT engagement is minimal, making CD155 necessary for TIGIT-mediated suppression and a plausible selector for tumors likely to respond to TIGIT-targeted therapy [139]. High tumor CD155 suppresses effector function via TIGIT and creates resistance to PD-1/CTLA-4 blockade; disrupting CD155–TIGIT restores T-cell activity, supporting CD155 as a mechanistic biomarker [140]. Early clinical data suggest higher CD155 may track with improved outcomes on combined TIGIT/PD-1 approaches, whereas CD155 alone predicts poorer response to PD-1 monotherapy [140,141].

Immune contexture signatures: Composite biomarkers incorporating multiple parameters T-cell infiltration, Treg prevalence, MDSC abundance, checkpoint expression may ultimately prove more predictive than any single marker. Machine learning approaches applied to multiplex immunohistochemistry or transcriptomic data are actively being developed [142,143].

#### 5.2.3. Preclinical Model Limitations

Traditional preclinical models inadequately recapitulate the human tumor microenvironment:

Immunodeficient mouse models lack adaptive immunity entirely and cannot model checkpoint inhibitor responses, as these therapies require functional T and B cells. While useful for engrafting human tumors, they necessitate “humanized” immune reconstitution to study immunotherapies effectively [144,145].

Syngeneic systems, despite utility for mechanistic immunology, incompletely model the genomic, microenvironmental, and phenotypic heterogeneity of human cancers that evolve over years. Implanted lines are comparatively homogeneous and selected for take and growth [146]. Even within the ID8 ovarian model, site and niche alter phenotype orthotopic, subcutaneous, ascites, and intestinal sites yield distinct epithelial–mesenchymal states and immune repertoires underscoring model-dependent variability that still falls short of patient-level diversity [147].

Cell line-based in vitro models cannot capture the multicellular complexity of the tumor microenvironment or the spatial organization essential for immune surveillance. Even advanced co-cultures often miss organized vasculature, metabolite gradients, and stromal–immune architecture that shape antigen presentation, trafficking, and exhaustion [148,149].

## 6. Future Directions and Unanswered Questions

As the field advances toward clinical implementation of TIGIT/PD-1 co-blockade in gynecologic malignancies, several critical questions remain. Addressing these uncertainties will determine whether the promising preclinical rationale translates into meaningful improvements in patient outcomes.

### 6.1. Optimal Sequencing and Combination Partners

The question of when to initiate TIGIT blockade concurrent with PD-1 inhibition or sequentially upon resistance carries significant therapeutic implications. Preclinical evidence strongly favors concurrent initiation: PD-1 blockade can induce compensatory TIGIT upregulation on tumor-reactive CD8+ T cells, and dual blockade additively restores proliferation, cytokine production, and degranulation in human tumor-infiltrating lymphocytes ex vivo and in murine models [83,109]. Mechanistically, co-blockade is necessary to fully restore CD226 signaling and optimize CD8+ T-cell responses, supporting concurrent rather than delayed initiation [103].

However, clinical validation of optimal timing remains incomplete. Early trials suggest promise for combination therapy, but definitive randomized data comparing concurrent versus sequential strategies are lacking. Thus, while preclinical data favor concurrent initiation, superiority over a sequential “on-resistance” approach remains unproven [13,104].

Beyond PD-1, identifying additional combination partners that maximize benefit represents a high-priority research direction. Rational candidates include:

Chemotherapy: Immunogenic cell death from agents such as oxaliplatin and taxanes can increase antigen release, dendritic cell activation, and PD-L1 modulation, enhancing checkpoint blockade. Clinical synergy has been observed particularly with oxaliplatin backbones in gastrointestinal cancers [150], and improved outcomes when immune checkpoint inhibitors are combined with taxanes in advanced non-small cell lung cancer across PD-L1 strata [151].

PARP inhibitors: In ovarian cancer, PARP inhibition causes DNA damage that activates the cGAS–STING pathway, driving type I interferon signaling and CD8+ T-cell infiltration. These effects are augmented by PD-1 blockade in BRCA1-deficient ovarian models [152]. Combinations of PARP inhibitors with PD-1 or TIGIT checkpoint blockade are under active study in gynecologic cancers, with the strongest signals to date in BRCA-mutated and homologous recombination-deficient ovarian disease [153].

Anti-angiogenic agents: Bevacizumab can normalize tumor vasculature and diminish immunosuppressive myeloid-derived suppressor cells, facilitating greater CD8+ T-cell infiltration and activity. In patients and preclinical models, bevacizumab lowered circulating MDSCs and increased intratumoral T cells, supporting synergy with PD-1/PD-L1 blockade [154]. In metastatic renal cell carcinoma and preclinical lung cancer, adding bevacizumab to checkpoint inhibitors increased CD8+ T-cell trafficking, effector gene programs, and cytotoxic function [155].

TGF-β inhibitors: In ovarian cancer, TGF-β signaling drives stromal barriers, immune exclusion, and Treg-mediated suppression. Mesenchymal and immune-excluded tumor states show elevated TGF-β programs that correlate with poor response to PD-1/PD-L1 therapy [156]. Preclinical data support that TGF-β blockade can restore CD8+ T-cell access and synergize with PD-1 inhibition, providing a rationale to combine TGF-β inhibitors with PD-1/TIGIT blockade in mesenchymal-subtype ovarian cancers [157].

### 6.2. Resistance Mechanisms to Dual Blockade

Even the most promising PD-1/TIGIT combinations will encounter resistance. Understanding these mechanisms is essential for developing third-line strategies and next-generation combination approaches.

Tumor-intrinsic mechanisms include defects in antigen presentation machinery such as β2-microglobulin and JAK1/2 loss-of-function mutations that impair interferon-γ signaling along with low tumor mutational burden resulting in paucity of neoantigens. Transcriptional programs driving immune exclusion and epithelial–mesenchymal transition, collectively termed “IPRES,” further limit checkpoint inhibitor efficacy [103,158].

Microenvironment-mediated resistance involves dominance of CD155 expression with concurrent downregulation of CD226 on T cells, enrichment of immunosuppressive M2 macrophages and Tregs, and altered cytokine signaling that blunts cytotoxicity. Adaptive escape includes upregulation of alternative checkpoints such as LAG-3, TIM-3, and CTLA-4, which sustain T-cell exhaustion despite PD-1/TIGIT blockade [159]. Loss of β2-microglobulin after initial response and increased CD155 expression through regulators like NECTIN4 further favor TIGIT engagement despite ongoing PD-1 inhibition [103,158].

Critical mechanistic convergence: Because PD-1 inhibits phosphorylation of CD226 and CD28 while TIGIT sterically blocks CD226-PVR interactions, insufficient CD226 signaling or DNAM-1 loss predicts failure of co-blockade. Additionally, metabolic competition high tumor glycolysis creating lactate-rich, glucose-poor microenvironments impairs effector T-cell function and promotes resistance [160]. The emergence of suppressive myeloid populations, including tumor-associated macrophages and MDSCs that resist TIGIT-axis modulation, maintains immunosuppression despite checkpoint therapy [161].

These interconnected resistance pathways underscore the need for third-line strategies targeting DNAM-1 axis restoration, antigen presentation enhancement, and myeloid reprogramming [103,158].

### 6.3. Development of Next-Generation TIGIT-Targeted Agents

Current TIGIT-targeting antibodies are primarily Fc-competent molecules designed to deplete TIGIT+ cells particularly regulatory T cells through antibody-dependent cellular cytotoxicity or phagocytosis. However, the relative contribution of Fc-mediated effector function versus pure checkpoint blockade to therapeutic efficacy remains actively debated [137].

Second-generation agents with optimized Fc engineering are entering clinical development. These include antibodies designed to selectively enhance or diminish Fc effector function depending on the desired mechanism of action preserving ADCC against TIGIT+ Tregs while avoiding depletion of activated TIGIT+ effector T cells. Bispecific formats, particularly PD-1/TIGIT bispecific antibodies, offer the potential for improved efficacy through coordinated checkpoint blockade and may provide convenience advantages over combination monoclonal antibody therapy [162].

Additional innovative approaches under investigation include antibody-drug conjugates targeting TIGIT-expressing cells, conditional bispecifics activated within the tumor microenvironment, and combination strategies incorporating cytokines or metabolic modulators to further enhance the activity of TIGIT/PD-1 co-blockade. The evolution of these next-generation agents will likely expand the therapeutic arsenal and address limitations of current first-generation combinations.

## 7. Conclusions

The modest efficacy of PD-1/PD-L1 monotherapy in gynecologic cancers reflects parallel, compensatory immunosuppressive mechanisms within the tumor microenvironment. TIGIT has emerged as a master checkpoint that coordinately regulates CD8+ T-cell exhaustion, NK-cell dysfunction, and Treg-mediated suppression, making it an ideal partner for PD-1 blockade. Preclinical evidence demonstrates potent synergy, and early-phase trials are actively evaluating this combination across gynecologic malignancies. However, realizing this potential requires overcoming substantial translational challenges spatial and temporal heterogeneity, biomarker limitations, and preclinical model inadequacies. An integrative framework combining advanced immunoprofiling (spatial transcriptomics, single-cell analysis) with patient-derived models (immuno-organoids, humanized mice) and rationally designed biomarker-driven trials offers the most promising path forward. By embracing TME complexity and developing mechanistically informed combination strategies, the field can move beyond the limitations of single-agent checkpoint blockade toward durable, personalized immunotherapy for women with gynecologic malignancies.

## Figures and Tables

**Table 1:** Comparative Immunological Landscape of Gynecologic Malignancies.

Feature	Ovarian Cancer (HGSOC)	Endometrial Cancer (MSI-H)	Endometrial Cancer (MSS)	Cervical Cancer
TME Classification	Immune-excluded/Desert [49]	Inflamed [51]	Immune-desert [51]	Inflamed [52]
Mutational Burden	Low–intermediate [53]	Very high [54]	Low [54]	Low (viral-driven) [55]
CD8+ T-cell Infiltration	Moderate, stromal-restricted [56]	High, intraepithelial [57]	Low [57]	High, intraepithelial [58,59]
Treg Prevalence	High (FoxP3+CD45RA−) [60]	Moderate [61]	Low-moderate [62]	High [63]
MDSC Accumulation	High (both subsets) [64]	Moderate [65]	High [65]	Moderate [65]
TIGIT Expression on CD8+ T cells	High on exhausted subsets [66,67]	High on exhausted subsets [66]	Moderate [66]	High on exhausted subsets [66]
TIGIT+ Treg Prevalence	High [25]	Moderate [25,28]	Low-moderate [28]	High [25]
PD-L1 Expression (Tumor Cells)	Inducible, focal [68,69]	Diffuse, constitutive [70,71]	Variable [72,73]	Diffuse, constitutive [74]
Dominant Suppressive Mechanism	Myeloid suppression, Treg activity [75]	T-cell exhaustion [76]	Immune exclusion, myeloid suppression [77]	T-cell exhaustion, adaptive resistance [78]
ICI Monotherapy Response (anti-PD-1/PD-L1)	<10–15% [79,80]	40–50% [81,82]	<10–15% [81,82]	12–17% [83,84]

Abbreviations: HGSOC, high-grade serous ovarian carcinoma; MSI-H, microsatellite instability-high; MSS, microsatellite stable; TME, tumor microenvironment; Treg, regulatory T cell; MDSC, myeloid-derived suppressor cell; ICI, immune checkpoint inhibitor.

**Table 2:** Selected Ongoing Clinical Trials Evaluating PD-(L)1 and TIGIT Co-Blockade in Gynecologic Cancers.

Trial Identifier	Phase	Agent	Combination	Patient Population	Primary Endpoints	Status
NCT03563716	II	Tiragolumab	+ Atezolizumab	Recurrent ovarian, cervical, endometrial cancers	ORR, PFS, Safety	Active, recruiting [119]
NCT04354246	II	Tiragolumab	+ Atezolizumab + Chemotherapy	Recurrent platinum-sensitive ovarian cancer	ORR, PFS	Active, recruiting [120]
NCT03628677	I/II	AB154	+ AB122 (anti-PD-1)	Advanced solid tumors (gynecologic expansion)	Safety, MTD, RP2D	Active, not recruiting [121]
NCT04570839	II	Vibostolimab	+ Pembrolizumab	Various solid tumors (gynecologic cohorts)	ORR, DOR	Active, recruiting [122]
NCT05026606	II	BMS-986207	+ Nivolumab + Ipilimumab	Recurrent gynecologic cancers	ORR, Safety	Active, recruiting [123]
NCT05231122	I/II	EOS884448	+ Pembrolizumab	Advanced solid tumors	Safety, PK, PD	Active, recruiting [124]

Abbreviations: ORR, objective response rate; PFS, progression-free survival; MTD, maximum tolerated dose; RP2D, recommended phase 2 dose; DOR, duration of response; PK, pharmacokinetics; PD, pharmacodynamics.

## Data Availability

No new data were created or analyzed in this study. Data sharing is not applicable to this article.
